# Deciphering the Therapeutic Resistance in Acute Myeloid Leukemia

**DOI:** 10.3390/ijms21228505

**Published:** 2020-11-12

**Authors:** Carmelo Gurnari, Simona Pagliuca, Valeria Visconte

**Affiliations:** 1 Department of Translational Hematology and Oncology Research, Taussig Cancer Institute, Cleveland Clinic, Cleveland, OH 44195, USA; carmelogurnari31@gmail.com (C.G.); smnpag@gmail.com (S.P.); 2 Department of Biomedicine and Prevention, University of Rome Tor Vergata, 00133 Rome, Italy

**Keywords:** acute myeloid leukemia, chemotherapy resistance, hypomethylating agent resistance

## Abstract

Acute myeloid leukemia (AML) is a clonal hematopoietic disorder characterized by abnormal proliferation, lack of cellular differentiation, and infiltration of bone marrow, peripheral blood, or other organs. Induction failure and in general resistance to chemotherapeutic agents represent a hindrance for improving survival outcomes in AML. Here, we review the latest insights in AML biology concerning refractoriness to therapies with a specific focus on cytarabine and daunorubicin which still represent milestones agents for inducing therapeutic response and disease eradication. However, failure to achieve complete remission in AML is still high especially in elderly patients (40–60% in patients >65 years old). Several lines of basic and clinical research have been employed to improve the achievement of complete remission. These lines of research include molecular targeted therapy and more recently immunotherapy. In terms of molecular targeted therapies, specific attention is given to *DNMT3A* and *TP53* mutant AML by reviewing the mechanisms underlying epigenetic therapies’ (e.g., hypomethylating agents) resistance and providing critical points and hints for possible future therapies overcoming AML refractoriness.

## 1. Introduction

Although the recent approval of several new pharmacologic agents contributed to expanding the panoply of options in the arena of acute myeloid leukemia (AML) treatments, the outcomes of resistant diseases are still dismal with only less than 30% of patients becoming long-term survivors [1,2]. In particular, if signs of progress have been made in terms of therapeutic support, anti-infectious prophylaxis, or choice of less intensive treatments for elderly patients (e.g., hypomethylating agents [HMA]), leading to the improvement of treatment-related mortality during induction therapy (decreased from 16% in the early 90′ to around 4–5% in the more recent years), the rates of patients with chemoresistant AML, (accounting for 35% to 45% of new cases) remain still virtually unchanged over the last few decades [3,4]. Thus, the acquisition of new insights in AML biology represents an unmet need in order to expand the horizon of targetable mechanisms and overcome therapeutic resistance in a disease responsible for the highest percentage of leukemic deaths [2]. The recent applications of genomic-scanning technologies (whole genome sequencing), combining high sensitivity and depth, have helped understanding several targetable mechanisms and/or mutated clones refractory to conventional therapies. Heterogeneity of subclones expanding over the course of therapies or appearance of new mutations has been attributed to treatment failure or refractoriness. Therefore, cytotoxic therapies failed to eliminate these therapy-resistant subclones. For instance, AML with *FLT3* mutations is an example of a subtype in which treatment with *FLT3* inhibitors, although promising, is not completely efficient due to the activation of target-dependent mechanisms (acquisition of point mutations in the kinase domain) reducing enzyme-inhibitor binding or through target-independent mechanisms and leading to primary or acquired resistance. Therapeutic resistance is one of the most crucial milestones in drug treatment. Translational research has indeed focused for years on the study of this topic. A variety of biological and genetic factors has been investigated with the recent involvement of untranscribed RNA products called non-coding RNAs (ncRNAs) in the regulation of main molecular drivers of AML (*CEBPA*, *FLT3*, *NPM1*) [5]. More recently efforts in drug development have produced small molecule inhibitors overcoming the adaptive resistance mechanism in *FLT3* mutant AML. In fact, *FLT3* mutant AML cells treated with FLT3 inhibitors (AC220, quizartinib), activate an innate immune pathway via the interleukin-1 receptor-associated kinase 1 and 4 (IRAK1/4). Drug design strategies were able to identify small molecules simultaneously inhibiting FLT3 and IRAK1/4 kinases and ultimately eliminating the adaptive resistance generated by this activation [6].

## 2. Mechanisms of Therapeutic Resistance

### 2.1. Chemotherapeutic Agents

**Figure 1:**
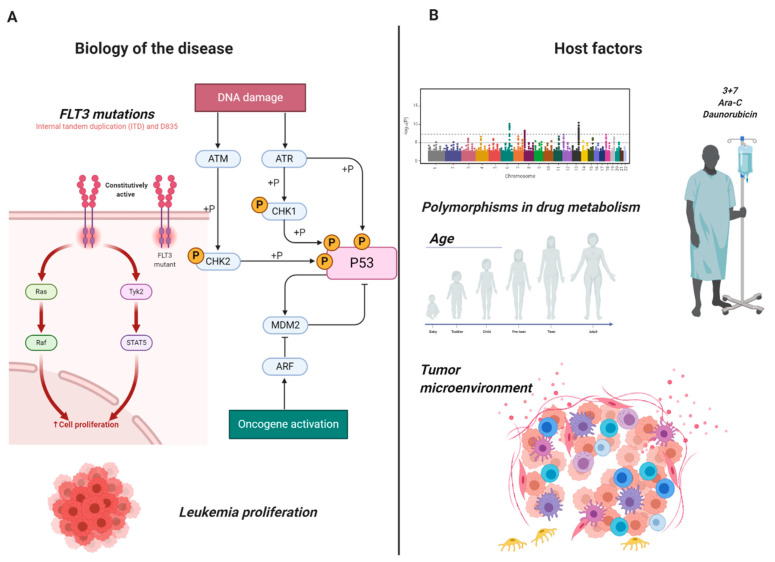
Mechanisms of chemoresistance in AML. Examples of the heterogeneity of mechanisms causing chemoresistance in adult acute myeloid leukemia: (**A**) Biology of the disease: constitutive activation of FLT3 leading to hyperproliferation and oncogenic activation of p53 through DNA damage induction. (**B**) Host factors: allele polymorphism leading to resistance to daunorubicin and cytosine arabinoside (Ara-C) and tumor microenvironment. Images were generated using BioRender.

The “3 + 7” regimen combining daunorubicin (DNR) and cytarabine (Ara-C) is still the backbone of induction treatment for adult patients with AML. However, primary refractory diseases or induction failures (PIF), defined as the persistence of at least 5% blasts in the bone marrow (BM) of patients receiving 1 or 2 cycles of induction therapy [7], still represent the outcomes of one-third of AML cases and show an abysmal long and short-term prognosis. Moreover, patients achieving a complete response (CR) in the early phases of therapy may subsequently relapse later by acquiring a secondary resistance. Deep DNA and RNA-sequencing technologies have demonstrated heterogeneity of causes leading to chemorefractoriness. Indeed, analysis of the genetic and transcriptomic profiles of refractory subpopulations has shown a differential expression in several pathways involved in transcription/translation, metabolism, microenvironment, DNA-damage and cell cycle. Figure 1 highlights examples of two main mechanisms underlying chemorefractoriness: the biology of the disease: (i) the occurrence of point mutations in *FLT3* kinase domain, leading to constitutive activation and triggering cell proliferation and (ii) the oncogenic activation resulted from DNA damage activity on the phosphorylation of p53; host factors: (i) variation in alleles for specific genes coding for enzymes involved in drug metabolism (Ara-C + DNR) and (ii) regulation of the tumor microenvironment (leukemic stem cells, lymph nodes, spleen for AML) especially in the modulation of the immune system following bone marrow transplantation. Herein, we comprehensively describe both mechanisms.

#### 2.1.1. Biology of the Disease: Genetic and Epigenetic Heterogeneity

Cytogenetic abnormalities have been traditionally used to prognostically stratify patients with AML [8]. The third-millennium genomic scanning approach with new platforms for whole-genome sequencing paved the way for a new AML classification taking into account, together with more traditional cytogenetic data, also somatic mutations in newly discovered genes and epigenetic patterns influencing patients’ outcomes and possibly therapeutic responsiveness [7,9]. Altogether the incorporation of mutations and cytogenetics information in new risk scoring systems have tremendously helped in the classification of entities of previous cytogenetics-based categorizations, e.g., normal karyotype (NK)-AML accounting for about 45% of new cases and falling in the umbrella of “intermediate risk” [10]. As a matter of fact and as a confirmation of the utility of integrated conventional cytogenetics and mutational screening, the introduction of *NPM1* mutation and *FLT3* allelic ratio in the new AML guidelines ELN 2017 helped to better stratify some of the previously considered “intermediate risk” patients. Approximately 40% of NK-AML patients harbour mutations in class III receptor tyrosine kinase either as a result of internal tandem duplication (*FLT3*-ITD) or substitution of the aspartic acid at position 835 with a tyrosine (*FLT3*-TKD) [11,12]. Both mutations result in the upregulation of downstream signalling pathways creating a hyperproliferative phenotype. Studies have also clarified that although only the former has a clear impact on disease prognosis, the latter has a not well-defined role in disease characteristics in terms of therapeutic response and prognosis. The involvement of other gene networks representing downstream targets of FLT3 has been investigated to understand chemoresistance. For example, it has been shown that RUNX3 expression may influence Ara-C resistance in patients with *FLT3*-ITD mutations. Studies conducted in human leukemic K562 transduced with *FLT3*-ITD and profiled by gene expression showed the transcriptional induction of RUNX3 in this model. Moreover, the downregulation of RUNX3 expression in these cells increased the sensitivity to Ara-C [13]. In the recent years, many FLT3 inhibitors have been approved to overcome the genetic disadvantage of this AML category. However, overexpression of BCL-2 (oncogene B cell lymphoma 2) has been shown to confer resistance to FLT3-inhibitors [14]. Similarly, *FLT3-*ITD mutations are more resistant to venetoclax, a BCL-2 inhibitor, when used as a single agent [15] while the combination with FLT3 inhibitors showed a more durable response in mouse models [16]. Indeed, venetoclax alone shows no response in small subsets of *FLT3*-ITD patients. Usually, *FLT3*-ITD mutations emerged at relapse following venetoclax monotherapy suggesting a possible mechanism of resistance. In a patient-derived *FLT3*-ITD xenograft model, the combination of venetoclax and quizartinib, a second-generation FLT3 inhibitor, had a greater anti-tumor activity compared to quizartinib or venetoclax monotherapy. These studies also showed that to overcome this resistance, three proteins (BCL-2, BCL-XL and MCL-1) should be targeted at the same time by synergistically using quizartinib or venetoclax but not any other BCL-2 inhibitor (Table 1) [17].

The mechanisms of chemotherapeutic resistance seem to be quite variable across molecular subtypes and/or age of the patients. In pediatric patients indeed genetic mechanisms seem to be the drivers of chemoresistance. Of note is that, differently from the adult counterpart, in the pediatric population, AML *FLT3* gene mutations do not seem to be related to primary chemotherapeutic resistance while a subset of mutations in other genes such as *ASXL1*, *SETBP1* and *WT1* define specific groups of patients experiencing PIF [18]. In this study whole-genome DNA, transcriptome RNA and miRNA sequencing analyses were performed on pediatric AML experiencing PIF as part of the TARGET Data initiative. The authors pinpoint loss-of-function mutations of *KMT2C*, encoding a component of the myeloid/lymphoid or mixed-lineage leukemia (MLL) chromatin remodelling complex, as a possible marker of chemorefractoriness, phenocopying chromosome 7q deletion, frequently observed also in adult higher-risk myelodysplastic syndromes (MDS) and secondary AML and found to be particularly susceptible to epigenetic therapies [18,19]. The same genomic position of *KMT2C* (7q36.1) is shared with the *EZH2* gene, the histone methyltransferase enhancer of the Zeste Homolog 2, whose mutations are observed in up to 9% of de novo and secondary AML [20]. Loss of activity of this gene and lower protein levels have been linked to resistance to multiple drugs including Ara-C, for which *EZH2* mutated cell lines show a 5-fold decrease in sensitivity [21]. In fact, it has been demonstrated that in resistant cell lines as well as in primary cells from relapsed AML patients, EZH2 is hyperphopshorylated at Thr487 by the cyclin-dependent kinase 1 via heat shock protein 90 (HSP90) stabilization and ultimately degraded by the proteasome. Of note, the use of proteasome inhibitors, such as bortezomib, is able to restore Ara-C sensitivity in this context.

*DNMT3A*, mutated in approximately 30% of NK-AML and encoding a DNA methyltransferase that catalyses 5-methylcytosine methylation, is another example of mutation affecting prognosis, being related to anthracycline resistance and PIF [22]. The key function of *DNMT3A* resides in its regulatory domains which allow the interactions with histone methyltransferases. These histones modulate the expression of genes after therapies. The most common mutation in the *DNMT3A* gene occurs at position 882 (R882H). R882H mutation acts in a dominant-negative fashion to disrupt the de novo methyltransferase activity of normal homotetramer. Mutations in *DNMT3A* not only seem to co-occur with genomic alterations in *FLT3* and *NPM1* to induce leukemogenesis in mice but also to create DNR resistance through the blockage of DNA damage response mechanism initiated by checkpoint kinase 1 (an event necessary for DNR mechanism of action) [23].

*MLL* (mixed-lineage leukemia, now renamed Lysine [K]-specific MethylTransferase 2A, *KMT2A*) on chromosome 11q23 is mutated in up to 10% of adult AML cases and represents another peculiar example of gene defining an AML subtype with an exquisite chemorefractoriness [24]. MLL-rearrangements generate various chimeric proteins which ultimately confer leukemia-initiating activity to hematopoietic stem and progenitor cells [25]. These cytogenetic abnormalities are present in both pediatric and adult patients as well as therapy-related cases after topoisomerase II inhibitor exposure. MLL-rearranged AML has long-term survival rates ranging from less than 10% to approximately 50%, depending on the fusion partner gene, age at diagnosis and other risk factors. For example, the t(9;11)(p21.3;q23.3); MLLT3-KMT2A translocation has been assigned to the intermediate-risk group as per the ELN 2017 AML guidelines [7]. However, this is only one of several known translocations. A seminal study by Meyer et al. [26] provided a comprehensive analysis of the “MLL recombinome”, allowing the classification of the different MLL rearrangements and the analysis of their clinical associations. Recent studies have shown that CDK6 is a cell-cycle regulator and critical effector of MLL fusions and it is responsible for a myeloid differentiation block, making it an actionable target for overcoming the traditional drug resistance typical of MLL-rearranged AML [27]/Together with CDK6, also DOT1L, a histone methyltransferase involved in differentiation and proliferation of hematopoietic stem cells (HSCs), has been recently identified as a putative target in this AML subtype [28,29]. A particular note must be made also for the *FLT3* gene whose overexpression may be responsible for the poor prognosis of some MLL-rearranged AML cases, suitable for specific FLT3-inhibitors treatments [24].

Finally, *TP53* mutations, accounting for 5–10% of *de novo* AML and up to 30% of therapy-related cases, represent a model of aggressive disease because of treatment failure. *TP53* mutated AML patients show a characteristic genomic instability with a complex karyotype (CK) and various patterns of co-mutations according to the *TP53* allelic state, making this setting particularly prone to chemoresistance [30,31]. However, even patients without CK show a dismal outcome and treatment refractoriness, outlining the intrinsic potential of *TP53* signalling disruption as a pillar in determining leukemic cell survival and aggressiveness [32,33]. Although recent studies showed improved outcomes with the use of decitabine in *TP53* mutated AML and higher-risk MDS, responses are usually short-lasting and even patients achieving a morphological CR had next-generation sequencing (NGS) minimally residual disease (MRD) detectable clone(s) which later on were attributable to patients’ relapse [34]. Moreover, a recent study [35] demonstrated that a cohort of TCGA patients with available DNA sequencing data had around 3% of patients with detectable low-frequency *TP53* reads. These patients, characterized by lower levels of p21 expression, had also worse clinical outcome if compared to wild-type counterparts. In this study, the authors modelled this condition in vitro using two AML cell lines (OCI-AML2, MV4–11) bearing low-frequency single hotspot *TP53* mutations. Resistant cells from both cell lines expressed *TP53* mutations including the wild-type counterparts manifesting chemoresistance. Leukemic cells with *TP53* mutations at subclonal levels had a survival advantage because of higher fractions of leukemic stem cells (LSC) (as shown in these in vitro models) which ultimately led to chemoresistance. In particular, *TP53* mutation impaired GADD45A (growth arrest and DNA damage-inducible alpha) expression in resistant cells negatively affecting Ara-C responsiveness. In the same study, the authors also showed how romidepsin, an histone deacetylase inhibitor, may restore Ara-C sensitivity via elevation of p21/GADD45A expression [35].

#### 2.1.2. Host Factors

A large study conducted on 4601 patients with AML showed that age and performance status of the patient (host) together with other disease variables such as white blood cells (WBC) counts, disease ontogeny (primary vs. secondary), cytogenetics and *FLT3* and *NPM1* mutational status may predict therapy resistance [36]. Apart from classical host-related factors like age, invariably associated with the burden of comorbidities and the possibility of choice of more intensive treatments and allogeneic hematopoietic stem cell transplant (aHCT), specific patients’ characteristics have been related to chemorefractoriness [37]. Resistance to anthracyclines has been widely associated with overexpression of drug efflux pumps or polymorphisms in drug metabolism responsible for decreasing the therapeutic effect of chemotherapy agents. Multidrug resistance (MDR) efflux transporters of the ABC (ATP-binding cassette) family comprehend the P glycoprotein (P-gp) pump, encoded by the *ABCB1* gene, the multidrug resistance-associated protein 1 (MRP1, encoded by *ABCC1*) and the breast cancer resistance protein (BCRP, encoded by *ABCG2*) [38]. These ABC efflux pumps are able to extrude endogenous uncharged molecules such as cyclic nucleotides or leukotrienes but also cytotoxic drugs including Ara-C, *Vinca* alkaloids and epipodophyllotoxins, in the attempt to defend HSCs [39,40]. Differently from P-gp pumps, MRP1 is specific for organic anions while BCRPs efflux substances such as mitoxantrone, methotrexate and others [41,42]. Studies conducted in murine models, in which EZH2 function was suppressed by using a mutant of EZH2 lacking the catalytic SET domain subsequently transduced, showed that ABCG2 transporter family of gene was upregulated in EZH2-dSET-transduced cells. ABCG2 was identified as a new target of the PRC2 complex. More importantly ABCG2 is a stem cell marker and drug efflux transporter rendering cancer cells resistant to chemotherapeutic drugs [43]. Overexpression of ABC P-gp transporters, has also been shown to be related to *FLT3*-ITD and not only to *EZH2* mutations, possibly driving refractory to chemotherapeutic agents and depressing their sensitivity several hundred times [21,44,45].

Although a recent study of the Spanish PETHEMA (*Programa para el Tratamiento de Hemopatias Malignas*) group showed no differences in terms of single nucleotide polymorphism of *ABCB1* gene upon the efficacy of induction chemotherapy in AML, there was a significant association with induction death and ABCB1 triple variant haplotype [46]. Another study of the same group showed instead how cytarabine pathway polymorphisms influence response to induction treatment [47]. In particular, the authors studied the impact of 10 different polymorphisms in Ara-C metabolic pathway genes in a cohort of 225 adult patients with *de novo* AML showing the association of activating (deoxycytidine kinase, DCK) and deactivating (cytidine deaminase, CDA) genes variants with PIF [47]. As a matter of fact, increased levels of CDA are associated with Ara-C resistance in AML [48,49]. Moreover, in vitro studies showed a higher level of CDA activity in Ara-C resistant AML cell lines and demonstrated also the negative impact on the CDA A79C polymorphism [50]. Recently, EPHX1 (microsomal epoxide hydrolase 1), encoding for a genotoxic epoxides detoxifying enzyme, has been linked to susceptibility of AML cells to anthracycline treatment via regulation of cytochrome P450 isophormes (CYP1A1) and other drug-metabolizing enzymes (such as glutathione S-transferase) and apoptotic signaling (BCL-2) [51].

Finally, an important role in chemotherapy resistance is also played by the BM “niche”. Additionally known as BM “microenvironment” and first proposed by Schofield in 1978 [52], the niche compartment (divided into vascular and endosteal) contains all the mesenchymal stem and progenitor cells, endothelial, osteolineage, neuronal, immune cells and adipocytes, responsible for a *milieu* of secretory cytokines regulating all the biological functions of HSCs [53,54]. BM niche mediates chemoresistance with various mechanisms either via soluble factors or via cell adhesion mediation. For example, the vascular endothelial growth factor C (VEGF-C) rescues blasts from chemotherapeutic-induced apoptosis [55]. Similarly, osteoblasts protect leukemic cells from CXCL12-induced death via secretion of soluble factors [56] and also via cell adhesion mechanisms ensuring an “oasis in the desert” by preserving leukemic cells from chemotherapeutic agents arriving from the bloodstream [57]. Recently, it has been shown that AML cells are able to transform the BM niche into a leukemia growth-permissive and normal hematopoiesis-suppressive microenvironment through various mechanisms including exosome secretion via DKK1 expression (a suppressor of normal hematopoiesis and osteogenesis) and subsequent osteoblast loss [58,59]. Moreover, AML cells are responsible for the remodeling of the vasculature of central and endosteal BM regions via secretion of pro-inflammatory and anti-angiogenic cytokines. This secretion tends to impoverish the BM niche of stromal and osteoblastic cells generating a “pro-leukemic microenvironment” not able to support normal hematopoiesis [60].

Apart from soluble factors, also physical characteristics of BM niches such as hypoxia or acidic pH provide a protective microenvironment for leukemic blasts [61]. While hypoxia maintains leukemic cells quiescent reducing their drug sensitivity, which is oftentimes cell cycle phases dependent, higher levels of hypoxia-inducible factor 1 a (HIF1a) decrease glucose metabolism and increase lactate production, ultimately promoting blast survival [62,63]. This shift to aerobic glycolysis (responsible for the so-called Warburg effect) is the main metabolic alteration in cancer cells. For instance, in AML *FLT3*-ITD mutations generate a microenvironment promoting the Warburg effect via activation of protein kinase b (AKT) signaling. As a result, FOXO proteins translocate from nucleus to cytoplasm lowering cell metabolic activities and generating an overexpression of various ABC family transporters [64,65,66,67]. Finally, it has also been reported that the inflammatory response and the ferritin levels of the individual patients may play a role in predicting survival outcomes and in mediating chemorefractoriness in AML [68,69]. In particular, Bertoli et al. demonstrated that the gene signature of hyperferritinemic AML patients was enriched in genes of NF-kB, oxidative stress and iron and ferritin levels correlated with Ara-C refractoriness [69].

#### 2.1.3. Other Mechanisms

Several other mechanisms of chemoresistance are not related to gene mutations or polymorphisms of detoxifying enzymes. In the last few years, new insights into cancer research paved the way for alternative mechanisms of resistance to pharmacologic agents in AML biology.

Ubiquitin and SUMO proteins control a wide range of cellular functions at a posttranslational level [70] and their altered expression has been related to cancers in general and hematological malignancies [71,72]. Gatel and colleagues showed that the study of the proteomic signatures of resistant AML, in particular the SUMOylation and ubiquitination of critical enzymes involved in cell survival activities, may predict DNR and Ara-C sensitivity [73]. They selectively identified specific enzymes in the SUMO/ubiquitin pathways found deregulated in chemoresistant AML cell lines, making them suitable for becoming biomarkers of chemosensitivity.

Novel mechanisms of mediating resistance to anticancer drug activities have been introduced by the combination of microarrays/RNA-sequencing and bioinformatics. Long non-coding RNA (lncRNA) are unique RNA transcripts that play a pivotal role in cancer development and also in conferring drug resistance [74,75]. Having a secondary and tertiary structure they can exert protein functions [76] promoting chemoresistance through different mechanisms and by influencing splicing and epigenetics. Several lncRNA have been identified as oncogenes (RUNXOR and TUG1 among others) or tumor suppressors (CASC15, IRAIN) [77]. For instance, knockdown of lncHOXA-AS2 is able to re-sensitize AML cells to anthracyclines via the miR-520c-3p/S100A4 axis [78]. Moreover, lncRNA TUG1 is upregulated in anthracylines resistant AML cells via inhibition of miR-34a and recruitment of EZH2 or RUNXOR is upregulated in t(8;21) AML and after ARA-C treatment [79,80]. Finally, lncRNA UCA1 has been related to chemorefractoriness in pediatric AML via inhibition of the Warburg effect through miR-125a/hexokinase 2 (HK2) pathway [81].

#### 2.1.4. New Formulations of Old Therapies Overcoming Chemoresistance: The Case of CPX-351 and Nanoscale Delivery Systems

Targeting AML metabolism has been the most recent field of research in order to optimize drug delivery systems. After the theorization of the “Combination Index”, initially proposed by Chou-Talalay in order to quantitatively outline drug antagonism, synergism and additive effects, many efforts have been made to specifically find drug formulations with the best pharmacokinetics and pharmacodynamics properties [82]. In particular, liposomal formulations have been used to potentially enhance efficacy maintaining a fixed drug ratio, a longer half-life and evading the first-pass metabolism. CPX-351 is the fruit of a radiometric approach which is able to provide in a liposomal formulation a fixed 5:1 molar ratio of Ara-C and DNR within 100-nm-diameter liposomes, containing 1 mg of Ara-C and 0.44 mg of DNR for each unit [83,84]. Mice studies showed that intracellular concentrations of CPX-351 were maintained near the optimal ratio for as long as 1 day after infusion and that the drug was preferentially accumulated in the blast cells, ultimately providing a higher pharmacokinetic efficacy if compared to the standard “3+7” regimen [85,86,87]. During phase II studies, CPX-351 demonstrated better response rates in higher-risk AML such as AML-MRC (MDS-related changes) as well as secondary AML cases [88,89]. In particular, overall response rates (ORR) were higher in patients with adverse cytogenetics (ORR, 77.3% vs. 38.5%; *p* = 0.03) and secondary AML (57.6% vs. 31.6%; *p* = 0.06) with also a better overall survival in the latter group (12.1 months vs. 6.1 months; *p* = 0.01) [90]. Nanoscale drug delivery systems have grown considerably, displaying promising results in improving the delivery of biomolecules. More recently, studies have pointed out the possible involvement of exosome as nanoplatforms in drug delivery. In the past decade, “smart” drug delivery strategies have employed nano-technologies (nanoparticle-based drug-delivery systems) to improve drug solubility, stability and bioavailability, toxicity by narrowing therapeutic index, and more importantly chemoresistance. Polymeric nanoparticles (poly-D,L-lactadie-co-glycolide, PLGA) were used to encapsulate ATRA (all-trans retinoic acid), folate and retinoic acid grafted/dextran to deliver doxorubicin, or dendrimers to deliver cytarabine triphosphate [91].

Furthermore, exosomes are extracellular vesicles of 30–100 nm in diameter released from body fluids with the function of delivering signaling molecules to distant cells. These vesicles serve as natural nanocarriers given the possibility to manipulate their structure for clinical use. They have been reported elevated in cancer [92] and reduced after chemotherapy in AML patients [93]. Being able to create a cross-talk at a body level and to easily reach anatomic sanctuary like the central nervous system, exosomes may be used for special scenarios of patients with refractory disease also at an extramedullary level.

### 2.2. Hypomethylating Agents

Hypomethylating agents (HMA) such as 5-azacytidine (AZA) and decitabine (DEC) represent a breakthrough in the treatment of patients with MDS and AML. However, despite their efficacy in ameliorating transfusion dependency and survival outcomes, HMA alone cannot completely cure the disease and their efficacy, even after achieving CR, sooner or later is lost if not consolidated with other approaches such as aHCT [94,95]. Both AZA and DEC are pro-drugs processed by the pyrimidine metabolism and then incorporated into the DNA during replication via degradation of DNA methyltransferase enzymes (DNMTs), ultimately leading to termination of malignant cell replication [96]. Therefore, it has begun imperative to better understand the mechanism of resistance to HMA and ameliorate the response outcomes of this set of patients.

Recently, it has been shown that the continuous exposure to HMA, resulting in nucleotide imbalances due to off-target inhibition of thymidylate synthase and ribonucleotide reductase by DEC and AZA respectively, generates with time a weaker DNMT1-depletion [97]. Moreover, as for Ara-C, also for DEC Qin et al. demonstrated that the efficacy is dependent on the alteration of transporters or metabolizing enzymes such as CDA and DCK [98]. In particular, DEC resistance was related to a heterozygous point mutation in codon 98 (ACA to AGA) in HL60 cell lines ultimately leading to lower levels of DCK and thus refractoriness. To circumvent the aforementioned metabolic differences, a new generation dinucleotide decitabine analog, guadecitabine, is now on study for its pharmacological profile less dependent on inter-individual variability. Another mechanism shown to be related to DEC resistance is the downregulation of miR-29c, whose expression was recently found deregulated in high-risk AML [99].

Resistance to AZA may be caused by genomic predisposition such as mutations, which make the clone intrinsically resistant, or epigenetic mechanisms. *ASXL1* and *EZH2* gene mutations do not rely on methylation to drive leukemia evolution and may generate subclones regardless of AZA treatment as well as some *DNMT3A* frameshift mutations [100]. Moreover, it has been shown that PU.1 expression is decreased in high-risk MDS and that AZA is able to restore its normal level ultimately allowing cellular differentiation [101]. Starting from the evidence of shorter OS and higher methylation levels in patients undergoing AZA presenting low PU-1 expression, the authors linked AZA resistance to alterations in PU.1 expression and thus cellular differentiation. Newer formulations of AZA, such as Acadesine, are also under study and are showing promising results also in AZA-resistant MDS/AML [102].

## 3. Conclusions and Future Perspectives

**Figure 2:**
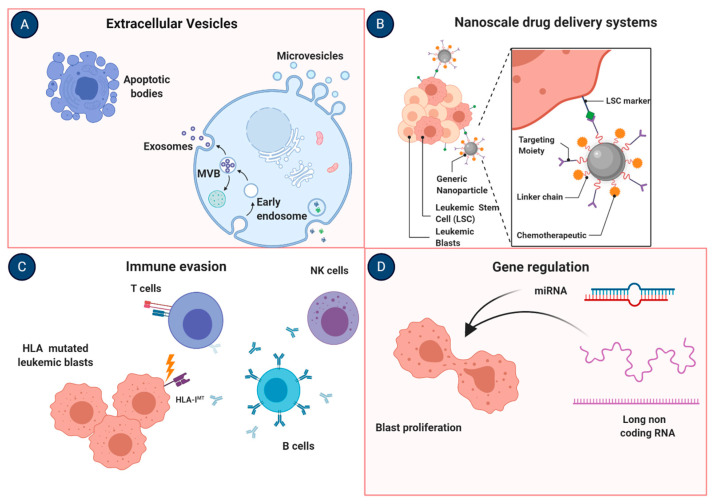
Actionable mechanisms to overcome chemoresistance in AML. (**A**) Exosomes are extracellular vesicles of 30–100 nm in diameter released from body fluids serving as natural nanocarriers with the possibility to manipulate their structure for clinical use. (**B**) Nanomedicine comprehends a variety of different nanoscale drug delivery systems possibly useful for better delivery of chemotherapy agents. (**C**) Human leukocyte antigen (HLA) machinery disruption via genetic (mutations) or epigenetic (downmodulation of the expression) mechanisms may lead to immune system evasion. (**D**) The study of gene regulation via miRNA or lncRNA may represent a useful tool to identify biomarkers of chemosensitivity being promising as also potential therapeutic targets. Images were generated using BioRender.

Patients achieving CR and subsequently relapsing after therapy or aHCT represent another difficult clinical scenario where other mechanisms may play a major role. The genomic and transcriptomic profile of AML has clarified that AML is a disease characterized by heterogeneity of factors where molecular networks often interplay with the tumor microenvironment. Given this heterogeneity, identified by the diverse subtypes of AML in the World Health Organization classification of AML and myeloid neoplasms in general, it is conceivable that precision medicine is the frontier in AML treatment. Individualized therapies are only possible to invent or re-discover, based on the consideration that a unique therapy will not apply to all AML population or “one size does not fit all”. The current rate of failure in conventional therapies or relapses and the high mortality rates of patients failing those regimens instruct us to consider all avenues of drug-based research to reduce chemoresistance. As a proof-of-concept of the variety of actionable roads of research attenuating chemoresistance and new modalities and systems improving drug metabolisms and targeting, we created a picture in Figure 2. Last but not least, the modulation of immunome and tumor microenvironment shows tremendous power in many cancers. Of note, the evasion from immunological pressure via human leukocyte antigen (HLA) machinery disruption or NK alloreactivity loss warrants future research and may represent actionable mechanisms for newer therapeutic targets along with studies highlighting the role of immunotherapy in combination with conventional treatments and their effects on the tumor microenvironment (Table 2).

## Figures and Tables

**Table 1:** Current ongoing clinical trials on AML targeting MDM2 and MCL-1 pathways.

Drug	Clinical Trial	Indication
***MDM2 inhibitors***		
Idasanutlin + cytarabine versus cytarabine only	NCT02545283	R/R AML
Venetoclax + Cobimetinib or Idasanutlin	NCT02670044	R/R AML Ineligible for Cytotoxic Therapy
MK-8242 + cytarabine	NCT01451437	R/R AML
HDM201+ cytarabine/anthracyclines	NCT03760445	R/R or Newly Diagnosed AML
***MCL-1 inhibitors***		
S64315	NCT02979366	AML and MDS
Venetoclax + S64315	NCT03672695	AML
AMG176	NCT02675452	R/R Multiple Myeloma and R/R AML
AMG397	NCT03465540	Multiple Myeloma, NHL, AML
AZD5991	NCT03218683	R/R Hematologic Malignancies including AML

R/R, relapsed/refractory; AML, acute myeloid leukemia; MDS, myelodysplastic syndrome; NHL, Non-Hodgkin lymphoma.

**Table 2:** Current ongoing clinical trials on AML with the use of immunologic agents.

Immunotherapy		
Drug	Clinical Trial	Indication
CD123 directed CAR-T cells	NCT02159495	R/R AML and Persistent/Recurrent Blastic Plasmacytoid Dendritic Cell Neoplasm
CYAD-01	NCT03018405	Multiple Cancer Indications including AML
CAR-T	NCT03190278	R/R AML
CD33 CAR	NCT03126864	R/R CD33-Positive AML
Nivolumab + azacitidine	NCT02532231	AML in Remission at High Risk for Relapse
Nivolumab + oral cyclophosphamide	NCT03417154	R/R AML and high-risk MDS
Pembrolizumab	NCT02768792	R/R AML
Ipilimumab	NCT02890329	R/R AML or MDS

R/R, relapsed/refractory; AML, acute myeloid leukemia; MDS, myelodysplastic syndrome.

## References

[1] Song X., Peng Y., Wang X., Chen Y., Jin L., Yang T., Qian M., Ni W., Tong X., Lan J. (2018). Incidence, Survival, and Risk Factors for Adults with Acute Myeloid Leukemia Not Otherwise Specified and Acute Myeloid Leukemia with Recurrent Genetic Abnormalities: Analysis of the Surveillance, Epidemiology, and End Results (SEER) Database, 2001–2013. Acta Haematol..

[2] Shallis R.M., Wang R., Davidoff A., Ma X., Zeidan A.M. (2019). Epidemiology of acute myeloid leukemia: Recent progress and enduring challenges. Blood Rev..

[3] Appelbaum F.R., Gundacker H., Head D.R., Slovak M.L., Willman C.L., Godwin J.E., Anderson J.E., Petersdorf S.H. (2006). Age and acute myeloid leukemia. Blood.

[4] Othus M., Kantarjian H., Petersdorf S., Ravandi F., Godwin J., Cortes J., Pierce S., Erba H., Faderl S., Appelbaum F.R. (2014). Declining rates of treatment-related mortality in patients with newly diagnosed AML given ‘intense’ induction regimens: A report from SWOG and MD Anderson. Leukemia.

[5] Zebisch A., Hatzl S., Pichler M., Wölfler A., Sill H. (2016). Therapeutic Resistance in Acute Myeloid Leukemia: The Role of Non-Coding RNAs. Int. J. Mol. Sci..

[6] Melgar K., Walker M.M., Jones L.M., Bolanos L.C., Hueneman K., Wunderlich M., Jiang J.-K., Wilson K.M., Zhang X., Sutter P. (2019). Overcoming adaptive therapy resistance in AML by targeting immune response pathways. Sci. Transl. Med..

[7] Döhner H., Estey E., Grimwade D., Amadori S., Appelbaum F.R., Büchner T., Dombret H., Ebert B.L., Fenaux P., Larson R.A. (2017). Diagnosis and management of AML in adults: 2017 ELN recommendations from an international expert panel. Blood.

[8] Döhner H., Estey E.H., Amadori S., Appelbaum F.R., Büchner T., Burnett A.K., Dombret H., Fenaux P., Grimwade D., Larson R.A. (2010). Diagnosis and management of acute myeloid leukemia in adults: Recommendations from an international expert panel, on behalf of the European LeukemiaNet. Blood.

[9] Papaemmanuil E., Gerstung M., Bullinger L., Gaidzik V.I., Paschka P., Roberts N.D., Potter N.E., Heuser M., Thol F., Bolli N. (2016). Genomic Classification and Prognosis in Acute Myeloid Leukemia. N. Engl. J. Med..

[10] Marcucci G., Mrózek K., Bloomfield C.D. (2005). Molecular heterogeneity and prognostic biomarkers in adults with acute myeloid leukemia and normal cytogenetics. Curr. Opin. Hematol..

[11] Gilliland D.G., Griffin J.D. (2002). The roles of FLT3 in hematopoiesis and leukemia. Blood.

[12] Abe M., Pelus L.M., Singh P., Hirade T., Onishi C., Purevsuren J., Taketani T., Yamaguchi S., Fukuda S. (2016). Internal Tandem Duplication in FLT3 Attenuates Proliferation and Regulates Resistance to the FLT3 Inhibitor AC220 by Modulating p21Cdkn1a and Pbx1 in Hematopoietic Cells. PLoS ONE.

[13] Damdinsuren A., Matsushita H., Ito M., Tanaka M., Jin G., Tsukamoto H., Asai S., Ando K., Miyachi H. (2015). FLT3-ITD drives Ara-C resistance in leukemic cells via the induction of RUNX3. Leuk. Res..

[14] Mehta S.V., Shukla S.N., Vora H.H. (2013). Overexpression of Bcl2 protein predicts chemoresistance in acute myeloid leukemia: Its correlation with FLT3. Neoplasma.

[15] Grundy M., Balakrishnan S., Fox M., Seedhouse C.H., Russell N.H. (2018). Genetic biomarkers predict response to dual BCL-2 and MCL-1 targeting in acute myeloid leukaemia cells. Oncotarget.

[16] Chyla B., Daver N., Doyle K., McKeegan E., Huang X., Ruvolo V., Wang Z., Chen K., Souers A., Leverson J. (2018). Genetic Biomarkers Of Sensitivity and Resistance to Venetoclax Monotherapy in Patients With Relapsed Acute Myeloid Leukemia. Am. J. Hematol..

[17] Mali R.S., Zhang Q., DeFilippis R., Cavazos A., Kuruvilla V.M., Raman J., Mody V., Choo E.F., Dail M., Shah N.P. (2020). Venetoclax combines synergistically with FLT3 inhibition to effectively target leukemic cells in FLT3-ITD+ acute myeloid leukemia models. Haematologica.

[18] McNeer N.A., Philip J., Geiger H., Ries R.E., Lavallée V.P., Walsh M., Shah M., Arora K., Emde A.K., Robine N. (2019). Genetic mechanisms of primary chemotherapy resistance in pediatric acute myeloid leukemia. Leukemia.

[19] Chen C., Liu Y., Rappaport A.R., Kitzing T., Schultz N., Zhao Z., Shroff A.S., Dickins R.A., Vakoc C.R., Bradner J.E. (2014). MLL3 is a haploinsufficient 7q tumor suppressor in acute myeloid leukemia. Cancer Cell.

[20] Lindsley R.C., Mar B.G., Mazzola E., Grauman P.V., Shareef S., Allen S.L., Pigneux A., Wetzler M., Stuart R.K., Erba H.P. (2015). Acute myeloid leukemia ontogeny is defined by distinct somatic mutations. Blood.

[21] Göllner S., Oellerich T., Agrawal-Singh S., Schenk T., Klein H.U., Rohde C., Pabst C., Sauer T., Lerdrup M., Tavor S. (2017). Loss of the histone methyltransferase EZH2 induces resistance to multiple drugs in acute myeloid leukemia. Nat. Med..

[22] Ley T.J., Ding L., Walter M.J., McLellan M.D., Lamprecht T., Larson D.E., Kandoth C., Payton J.E., Baty J., Welch J. (2010). DNMT3A Mutations in Acute Myeloid Leukemia. N. Engl. J. Med..

[23] Guryanova O.A., Shank K., Spitzer B., Luciani L., Koche R.P., Garrett-Bakelman F.E., Ganzel C., Durham B.H., Mohanty A., Hoermann G. (2016). DNMT3A mutations promote anthracycline resistance in acute myeloid leukemia via impaired nucleosome remodeling. Nat. Med..

[24] Winters A.C., Bernt K.M. (2017). MLL-Rearranged Leukemias-An Update on Science and Clinical Approaches. Front. Pediatr..

[25] Krivtsov A.V., Twomey D., Feng Z., Stubbs M.C., Wang Y., Faber J., Levine J.E., Wang J., Hahn W.C., Gilliland D.G. (2006). Transformation from committed progenitor to leukaemia stem cell initiated by MLL-AF9. Nature.

[26] Meyer C., Hofmann J., Burmeister T., Gröger D., Park T.S., Emerenciano M., Pombo de Oliveira M., Renneville A., Villarese P., Macintyre E. (2013). The MLL recombinome of acute leukemias in 2013. Leukemia.

[27] Placke T., Faber K., Nonami A., Putwain S.L., Salih H.R., Heidel F.H., Krämer A., Root D.E., Barbie D.A., Krivtsov A.V. (2014). Requirement for CDK6 in MLL-rearranged acute myeloid leukemia. Blood.

[28] Chen C.-W., Armstrong S.A. (2015). Targeting DOT1L and HOX gene expression in MLL-rearranged leukemia and beyond. Exp. Hematol..

[29] Klaus C.R., Iwanowicz D., Johnston D., Campbell C.A., Smith J.J., Moyer M.P., Copeland R.A., Olhava E.J., Scott M.P., Pollock R.M. (2014). DOT1L inhibitor EPZ-5676 displays synergistic antiproliferative activity in combination with standard of care drugs and hypomethylating agents in MLL-rearranged leukemia cells. J. Pharmacol. Exp. Ther..

[30] Bernard E., Nannya Y., Hasserjian R.P., Devlin S.M., Tuechler H., Medina-Martinez J.S., Yoshizato T., Shiozawa Y., Saiki R., Malcovati L. (2020). Implications of TP53 allelic state for genome stability, clinical presentation and outcomes in myelodysplastic syndromes. Nat. Med..

[31] Haase D., Stevenson K.E., Neuberg D., Maciejewski J.P., Nazha A., Sekeres M.A., Ebert B.L., Garcia-Manero G., Haferlach C., Haferlach T. (2019). TP53 mutation status divides myelodysplastic syndromes with complex karyotypes into distinct prognostic subgroups. Leukemia.

[32] Bowen D., Groves M.J., Burnett A.K., Patel Y., Allen C., Green C., Gale R.E., Hills R., Linch D.C. (2009). TP53 gene mutation is frequent in patients with acute myeloid leukemia and complex karyotype, and is associated with very poor prognosis. Leukemia.

[33] Welch J.S., Petti A.A., Miller C.A., Fronick C.C., O’Laughlin M., Fulton R.S., Wilson R.K., Baty J.D., Duncavage E.J., Tandon B. (2016). TP53 and Decitabine in Acute Myeloid Leukemia and Myelodysplastic Syndromes. N. Engl. J. Med..

[34] Montalban-Bravo G., Takahashi K., Garcia-Manero G. (2017). Decitabine in TP53-Mutated AML. N. Engl. J. Med..

[35] Yan B., Chen Q., Xu J., Li W., Xu B., Qiu Y. (2020). Low-frequency TP53 hotspot mutation contributes to chemoresistance through clonal expansion in acute myeloid leukemia. Leukemia.

[36] Walter R.B., Othus M., Paietta E.M., Racevskis J., Fernandez H.F., Lee J.W., Sun Z., Tallman M.S., Patel J., Gönen M. (2015). Effect of genetic profiling on prediction of therapeutic resistance and survival in adult acute myeloid leukemia. Leukemia.

[37] Yeung C.C.S., Radich J. (2017). Predicting Chemotherapy Resistance in AML. Curr. Hematol. Malig. Rep..

[38] Steinbach D., Legrand O. (2007). ABC transporters and drug resistance in leukemia: Was P-gp nothing but the first head of the Hydra?. Leukemia.

[39] Long L., Assaraf Y.G., Lei Z.-N., Peng H., Yang L., Chen Z.-S., Ren S. (2020). Genetic biomarkers of drug resistance: A compass of prognosis and targeted therapy in acute myeloid leukemia. Drug Resist. Updates.

[40] Marzac C., Garrido E., Tang R., Fava F., Hirsch P., De Benedictis C., Corre E., Lapusan S., Lallemand J.Y., Marie J.P. (2011). ATP Binding Cassette transporters associated with chemoresistance: Transcriptional profiling in extreme cohorts and their prognostic impact in a cohort of 281 acute myeloid leukemia patients. Haematologica.

[41] Borst P., Evers R., Kool M., Wijnholds J. (2000). A family of drug transporters: The multidrug resistance-associated proteins. J. Natl. Cancer Inst..

[42] Austin Doyle L., Ross D.D. (2003). Multidrug resistance mediated by the breast cancer resistance protein BCRP (ABCG2). Oncogene.

[43] Kawabata K.C., Hayashi Y., Inoue D., Meguro H., Sakurai H., Fukuyama T., Tanaka Y., Asada S., Fukushima T., Nagase R. (2018). High expression of ABCG2 induced by EZH2 disruption has pivotal roles in MDS pathogenesis. Leukemia.

[44] Nasilowska-Adamska B., Solarska I., Paluszewska M., Malinowska I., Jedrzejczak W.W., Warzocha K. (2014). FLT3-ITD and MLL-PTD influence the expression of MDR-1, MRP-1, and BCRP mRNA but not LRP mRNA assessed with RQ-PCR method in adult acute myeloid leukemia. Ann. Hematol..

[45] Breier A., Drobná Z., Docolomansky P., Barancik M. (2000). Cytotoxic activity of several unrelated drugs on L1210 mouse leukemic cell sublines with P-glycoprotein (PGP) mediated multidrug resistance (MDR) phenotype. A QSAR study. Neoplasma.

[46] Megías-Vericat J.E., Montesinos P., Herrero M.J., Moscardó F., Bosó V., Rojas L., Martínez-Cuadrón D., Hervás D., Boluda B., García-Robles A. (2017). Impact of ABC single nucleotide polymorphisms upon the efficacy and toxicity of induction chemotherapy in acute myeloid leukemia. Leuk. Lymphoma.

[47] Megías-Vericat J.E., Montesinos P., Herrero M.J., Moscardó F., Bosó V., Martínez-Cuadrón D., Rojas L., Rodríguez-Veiga R., Boluda B., Sendra L. (2017). Influence of cytarabine metabolic pathway polymorphisms in acute myeloid leukemia induction treatment. Leuk. Lymphoma.

[48] Lamba J.K. (2009). Genetic factors influencing cytarabine therapy. Pharmacogenomics.

[49] Levin M., Stark M., Berman B., Assaraf Y.G. (2019). Surmounting Cytarabine-resistance in acute myeloblastic leukemia cells and specimens with a synergistic combination of hydroxyurea and azidothymidine. Cell Death Dis..

[50] Medina-Sanson A., Ramírez-Pacheco A., Moreno-Guerrero S.S., Dorantes-Acosta E.M., Sánchez-Preza M., Reyes-López A. (2015). Role of Genetic Polymorphisms of Deoxycytidine Kinase and Cytidine Deaminase to Predict Risk of Death in Children with Acute Myeloid Leukemia. BioMed Res. Int..

[51] Cheng H., Huang C., Tang G., Qiu H., Gao L., Zhang W., Wang J., Yang J., Chen L. (2019). Emerging role of EPHX1 in chemoresistance of acute myeloid leukemia by regurlating drug-metabolizing enzymes and apoptotic signaling. Mol. Carcinogenes..

[52] Schofield R. (1978). The relationship between the spleen colony-forming cell and the haemopoietic stem cell. Blood Cells.

[53] He N., Zhang L., Cui J., Li Z. (2014). Bone Marrow Vascular Niche: Home for Hematopoietic Stem Cells. Bone Marrow Res..

[54] Wang A., Zhong H. (2018). Roles of the bone marrow niche in hematopoiesis, leukemogenesis, and chemotherapy resistance in acute myeloid leukemia. Hematology.

[55] Dias S., Choy M., Alitalo K., Rafii S. (2002). Vascular endothelial growth factor (VEGF)-C signaling through FLT-4 (VEGFR-3) mediates leukemic cell proliferation, survival, and resistance to chemotherapy. Blood.

[56] Kremer K.N., Dudakovic A., McGee-Lawrence M.E., Philips R.L., Hess A.D., Smith B.D., van Wijnen A.J., Karp J.E., Kaufmann S.H., Westendorf J.J. (2014). Osteoblasts Protect AML Cells From SDF-1-Induced Apoptosis. J. Cell. Biochem..

[57] Tabe Y., Konopleva M. (2014). Advances in understanding the leukaemia microenvironment. Br. J. Haematol..

[58] Kumar B., Garcia M., Weng L., Jung X., Murakami J.L., Hu X., McDonald T., Lin A., Kumar A.R., DiGiusto D.L. (2018). Acute myeloid leukemia transforms the bone marrow niche into a leukemia-permissive microenvironment through exosome secretion. Leukemia.

[59] Kumar B., Chen C.C. (2018). Acute myeloid leukemia remodels endosteal vascular niche into a leukemic niche. Stem Cell Investig..

[60] Duarte D., Hawkins E.D., Akinduro O., Ang H., De Filippo K., Kong I.Y., Haltalli M., Ruivo N., Straszkowski L., Vervoort S.J. (2018). Inhibition of Endosteal Vascular Niche Remodeling Rescues Hematopoietic Stem Cell Loss in AML. Cell Stem Cell.

[61] Drolle H., Wagner M., Vasold J., Kütt A., Deniffel C., Sotlar K., Sironi S., Herold T., Rieger C., Fiegl M. (2015). Hypoxia regulates proliferation of acute myeloid leukemia and sensitivity against chemotherapy. Leuk. Res..

[62] Lum J.J., Bui T., Gruber M., Gordan J.D., DeBerardinis R.J., Covello K.L., Simon M.C., Thompson C.B. (2007). The transcription factor HIF-1alpha plays a critical role in the growth factor-dependent regulation of both aerobic and anaerobic glycolysis. Genes Dev..

[63] Valsecchi R., Coltella N., Belloni D., Ponente M., Ten Hacken E., Scielzo C., Scarfò L., Bertilaccio M.T., Brambilla P., Lenti E. (2016). HIF-1α regulates the interaction of chronic lymphocytic leukemia cells with the tumor microenvironment. Blood.

[64] Burgering B.M., Kops G.J. (2002). Cell cycle and death control: Long live Forkheads. Trends Biochem. Sci..

[65] Gurnari C., Falconi G., De Bellis E., Voso M.T., Fabiani E. (2019). The Role of Forkhead Box Proteins in Acute Myeloid Leukemia. Cancers.

[66] Suda T., Arai F., Shimmura S. (2005). Regulation of stem cells in the niche. Cornea.

[67] Schaich M., Soucek S., Thiede C., Ehninger G., Illmer T., The SHG AML96 Study Group (2005). MDR1 and MRP1 gene expression are independent predictors for treatment outcome in adult acute myeloid leukaemia. Br. J. Haematol..

[68] Lebon D., Vergez F., Bertoli S., Harrivel V., De Botton S., Micol J.B., Marolleau J.P., Récher C. (2015). Hyperferritinemia at diagnosis predicts relapse and overall survival in younger AML patients with intermediate-risk cytogenetics. Leuk. Res..

[69] Bertoli S., Paubelle E., Bérard E., Saland E., Thomas X., Tavitian S., Larcher M.-V., Vergez F., Delabesse E., Sarry A. (2019). Ferritin heavy/light chain (FTH1/FTL) expression, serum ferritin levels, and their functional as well as prognostic roles in acute myeloid leukemia. Eur. J. Haematol..

[70] Streich F.C., Lima C.D. (2014). Structural and functional insights to ubiquitin-like protein conjugation. Annu. Rev. Biophys..

[71] Seeler J.S., Dejean A. (2017). SUMO and the robustness of cancer. Nat. Rev. Cancer.

[72] Boulanger M., Paolillo R., Piechaczyk M., Bossis G. (2019). The SUMO Pathway in Hematomalignancies and Their Response to Therapies. Int. J. Mol. Sci..

[73] Gâtel P., Brockly F., Reynes C., Pastore M., Hicheri Y., Cartron G., Piechaczyk M., Bossis G. (2020). Ubiquitin and SUMO conjugation as biomarkers of acute myeloid leukemias response to chemotherapies. Life Sci. Alliance.

[74] Jiang W., Xia J., Xie S., Zou R., Pan S., Wang Z.W., Assaraf Y.G., Zhu X. (2020). Long non-coding RNAs as a determinant of cancer drug resistance: Towards the overcoming of chemoresistance via modulation of lncRNAs. Drug Resist. Updates Rev. Comment. Antimicrob. Anticancer Chemother..

[75] Esteller M. (2011). Non-coding RNAs in human disease. Nat. Rev. Genet..

[76] Novikova I.V., Hennelly S.P., Tung C.S., Sanbonmatsu K.Y. (2013). Rise of the RNA machines: Exploring the structure of long non-coding RNAs. J. Mol. Biol..

[77] Gourvest M., Brousset P., Bousquet M. (2019). Long Noncoding RNAs in Acute Myeloid Leukemia: Functional Characterization and Clinical Relevance. Cancers.

[78] Dong X., Fang Z., Yu M., Zhang L., Xiao R., Li X., Pan G., Liu J. (2018). Knockdown of Long Noncoding RNA HOXA-AS2 Suppresses Chemoresistance of Acute Myeloid Leukemia via the miR-520c-3p/S100A4 Axis. Cell. Physiol. Biochem..

[79] Li Q., Song W., Wang J. (2019). TUG1 confers Adriamycin resistance in acute myeloid leukemia by epigenetically suppressing miR-34a expression via EZH2. Biomed. Pharmacother..

[80] Wang H., Li W., Guo R., Sun J., Cui J., Wang G., Hoffman A.R., Hu J.F. (2014). An intragenic long noncoding RNA interacts epigenetically with the RUNX1 promoter and enhancer chromatin DNA in hematopoietic malignancies. Int. J. Cancer.

[81] Zhang Y., Liu Y., Xu X. (2018). Knockdown of LncRNA-UCA1 suppresses chemoresistance of pediatric AML by inhibiting glycolysis through the microRNA-125a/hexokinase 2 pathway. J. Cell. Biochem..

[82] Chou T.C., Talalay P. (1984). Quantitative analysis of dose-effect relationships: The combined effects of multiple drugs or enzyme inhibitors. Adv. Enzym. Regul..

[83] Feldman E.J., Lancet J.E., Kolitz J.E., Ritchie E.K., Roboz G.J., List A.F., Allen S.L., Asatiani E., Mayer L.D., Swenson C. (2011). First-in-man study of CPX-351: A liposomal carrier containing cytarabine and daunorubicin in a fixed 5:1 molar ratio for the treatment of relapsed and refractory acute myeloid leukemia. J. Clin. Oncol. Off. J. Am. Soc. Clin. Oncol..

[84] Alfayez M., Kantarjian H., Kadia T., Ravandi-Kashani F., Daver N. (2020). CPX-351 (vyxeos) in AML. Leuk. Lymphoma.

[85] Krauss A.C., Gao X., Li L., Manning M.L., Patel P., Fu W., Janoria K.G., Gieser G., Bateman D.A., Przepiorka D. (2019). FDA Approval Summary: (Daunorubicin and Cytarabine) Liposome for Injection for the Treatment of Adults with High-Risk Acute Myeloid Leukemia. Clin. Cancer Res. Off. J. Am. Assoc. Cancer Res..

[86] Lim W.S., Tardi P.G., Dos Santos N., Xie X., Fan M., Liboiron B.D., Huang X., Harasym T.O., Bermudes D., Mayer L.D. (2010). Leukemia-selective uptake and cytotoxicity of CPX-351, a synergistic fixed-ratio cytarabine:daunorubicin formulation, in bone marrow xenografts. Leuk. Res..

[87] Kim H.P., Gerhard B., Harasym T.O., Mayer L.D., Hogge D.E. (2011). Liposomal encapsulation of a synergistic molar ratio of cytarabine and daunorubicin enhances selective toxicity for acute myeloid leukemia progenitors as compared to analogous normal hematopoietic cells. Exp. Hematol..

[88] Cortes J.E., Goldberg S.L., Feldman E.J., Rizzeri D.A., Hogge D.E., Larson M., Pigneux A., Recher C., Schiller G., Warzocha K. (2015). Phase II, multicenter, randomized trial of CPX-351 (cytarabine:daunorubicin) liposome injection versus intensive salvage therapy in adults with first relapse AML. Cancer.

[89] Ryan D.H., Uy G.L., Cortes J.E., Newell L.F., Ritchie E.K., Stuart R.K., Strickland S.A., Hogge D., Solomon S.R., Stone R.M. (2018). Efficacy and Safety of CPX-351 Versus 7+3 in a Subgroup of Older Patients with Newly Diagnosed Acute Myeloid Leukemia with Myelodysplasia-Related Changes (AML-MRC) Enrolled in a Phase 3 Study. Blood.

[90] Lancet J.E., Cortes J.E., Hogge D.E., Tallman M.S., Kovacsovics T.J., Damon L.E., Komrokji R., Solomon S.R., Kolitz J.E., Cooper M. (2014). Phase 2 trial of CPX-351, a fixed 5:1 molar ratio of cytarabine/daunorubicin, vs cytarabine/daunorubicin in older adults with untreated AML. Blood.

[91] Chen K.T.J., Gilabert-Oriol R., Bally M.B., Leung A.W.Y. (2019). Recent Treatment Advances and the Role of Nanotechnology, Combination Products, and Immunotherapy in Changing the Therapeutic Landscape of Acute Myeloid Leukemia. Pharm. Res..

[92] Melo S.A., Sugimoto H., O’Connell J.T., Kato N., Villanueva A., Vidal A., Qiu L., Vitkin E., Perelman L.T., Melo C.A. (2014). Cancer exosomes perform cell-independent microRNA biogenesis and promote tumorigenesis. Cancer Cell.

[93] Hong C.-S., Muller L., Whiteside T.L., Boyiadzis M. (2014). Plasma Exosomes as Markers of Therapeutic Response in Patients with Acute Myeloid Leukemia. Front. Immunol..

[94] Kadia T.M., Jabbour E., Kantarjian H. (2011). Failure of hypomethylating agent-based therapy in myelodysplastic syndromes. Semin. Oncol..

[95] Fenaux P., Mufti G.J., Hellstrom-Lindberg E., Santini V., Finelli C., Giagounidis A., Schoch R., Gattermann N., Sanz G., List A. (2009). Efficacy of azacitidine compared with that of conventional care regimens in the treatment of higher-risk myelodysplastic syndromes: A randomised, open-label, phase III study. Lancet Oncol..

[96] Saunthararajah Y. (2013). Key clinical observations after 5-azacytidine and decitabine treatment of myelodysplastic syndromes suggest practical solutions for better outcomes. Hematol. Am. Soc. Hematol. Educ. Program.

[97] Gu X., Tohme R., Tomlinson B., Sakre N., Hasipek M., Durkin L., Schuerger C., Grabowski D., Zidan A.M., Radivoyevitch T. (2020). Decitabine- and 5-azacytidine resistance emerges from adaptive responses of the pyrimidine metabolism network. Leukemia.

[98] Qin T., Jelinek J., Si J., Shu J., Issa J.P. (2009). Mechanisms of resistance to 5-aza-2′-deoxycytidine in human cancer cell lines. Blood.

[99] Tang L.-J., Sun G.-K., Zhang T.-J., Wu D.-H., Zhou J.-D., Ma B.-B., Xu Z.-J., Wen X.-M., Chen Q., Yao D.-M. (2019). Down-regulation of miR-29c is a prognostic biomarker in acute myeloid leukemia and can reduce the sensitivity of leukemic cells to decitabine. Cancer Cell Int..

[100] Minarik L., Drusbosky L.M., Abbasi T., Vargova K., Kulvait V., Jonasova A., Singh N.K., Kumar C., Suseela R.P., Patil M. (2018). Mechanisms of Azacitidine Chemotherapy Resistance in AML and MDS and New Therapy Options. Blood.

[101] Curik N., Burda P., Vargova K., Pospisil V., Belickova M., Vlckova P., Savvulidi F., Necas E., Hajkova H., Haskovec C. (2012). 5-azacitidine in aggressive myelodysplastic syndromes regulates chromatin structure at PU.1 gene and cell differentiation capacity. Leukemia.

[102] Cluzeau T., Furstoss N., Savy C., El Manaa W., Zerhouni M., Blot L., Calleja A., Dufies M., Dubois A., Ginet C. (2019). Acadesine Circumvents Azacitidine Resistance in Myelodysplastic Syndrome and Acute Myeloid Leukemia. Int. J. Mol. Sci..

